# Inhibitory effects and amino acid metabolism regulations of active polyphenol from foxtail millet bran on chronic colitis in mice

**DOI:** 10.3389/fnut.2025.1714755

**Published:** 2025-12-18

**Authors:** Ruipeng Yang, Shuiling He, Jingli Wang, Jieya Yang, Ruijun Su, Wenjing Zhao

**Affiliations:** 1 Biological Science and Technology College, Taiyuan Normal University, Jinzhong, China; 2 College of Biology, Hunan University, Changsha, China; 3 Department of Landscape Architecture, Shanxi Forestry Vocational Technical College, Taiyuan, China

**Keywords:** foxtail millet bran, polyphenol, active components, chronic colitis, non-target metabolomics, amino acid metabolism

## Abstract

**Introduction:**

Inflammatory bowel disease (IBD) is frequently associated with metabolic imbalances. Polyphenols have demonstrated efficacy in alleviating colitis by restoring the metabolic disorders. Our previous studies revealed that bound polyphenols extracted from millet bran could alleviate acute colitis and colitis-associated colorectal cancer (CRC) via restoring the gut microbiome and that the low molecular weight (MW) (<200 Da) portion of bound polyphenol (BPLP) constituted the primary active component, comprising six phenolic acids.

**Methods:**

To further evaluate the effects of BPLP on inflammation, a dextran sodium sulfateb(DSS)-induced experimental colitis model was constructed, and BPLP was gavaged on mice. The effects of BPLP on colitis were assessed by detecting the weight, mouse status, gut barrier integrity, and inflammatory cytokine secretion. Additionally, non-targeted metabolomics was used to identify altered metabolites.

**Results and discussion:**

BPLP administration restored body weight and colon length, protected epithelial structure from DSS-induced damage, and relieved chronic colitis. In colons, BPLP reduced the levels of pro-inflammatory cytokines (TNF-α, IL-6, and IL-1β), enhanced the secretion of the anti-inflammatory cytokine IL-10, and upregulated the expression of tight junction proteins. Nontarget metabolomic results showed that BPLP alleviated colitis by modulating amino acid metabolism pathways, including valine/leucine/isoleucine biosynthesis,phenylalanine/tyrosine/tryptophan biosynthesis, and phenylalanine metabolism. Furthermore, alterations in specific amino acids, such as valine and beta-alanine, were consistent with profiles observed in clinical IBD patients. Collectively, these results indicate that BPLP effectively alleviates chronic colitis in mice and regulates inflammation-related amino acid metabolism *in vivo*.

## Graphical abstract

**Figure:**
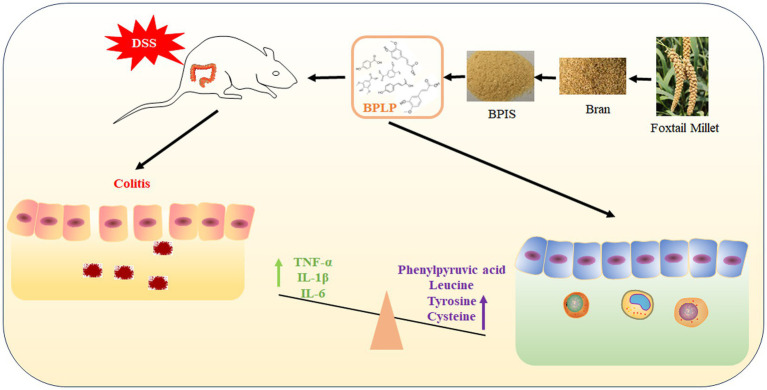

## Highlights

- BPLP extracted from foxtail millet bran relieved DSS-induced experimental colitis.
- BPLP may be a natural candidate for the development of a safe and effective adjuvant to improve IBD.
- The study supported the dietary recommendations proposed by nutritionists that increasing whole grain intake brings great public health benefits.

## 1 Introduction

Inflammatory bowel disease (IBD) is a chronic relapsing condition characterized by dysregulated mucosal immune responses and disrupted intestinal barrier functions in the gastrointestinal tract, primarily comprising Crohn’s disease (CD) and ulcerative colitis (UC) (1, 2). Accumulating evidence indicates that pathophysiological disturbances in IBD are reflected by alterations in metabolite profiles, particularly imbalances in amino acid metabolism (3, 4). A recent study demonstrated that amino acid-balanced diets derived from grains alleviated the colitis symptoms and altered the structure of gut microbiota by decreasing harmful bacteria (3). Additionally, the communication between the gut microbiome and amino acid metabolism (especially tyrosine and tryptophan metabolism) changed and played a pivotal role in affecting mice with UC (5). Nikolaus et al. (6) analyzed serum samples from over 500 IBD patients and identified that the deficiency of tryptophan exacerbated IBD or increased disease activity. Arginine metabolism has also been intimately associated with gut pathophysiology and directly influences the pathogenesis of IBD. Supplementation with arginine represents a promising therapeutic strategy for IBD tailored for different individuals at different disease stages (7). Xin et al. (8) reported distinct amino acid metabolic profiles in UC patients compared to healthy controls, suggesting that amino acid composition is related to the incidence of UC. These findings revealed the involvement of amino acid metabolism in the pathogenesis of inflammation and highlight the potential of targeting amino acids and their metabolites as an effective therapeutic approach for IBD.

As natural secondary metabolites derived from plants, polyphenols have been shown to possess antioxidant and anti-inflammatory activities (9). Numerous studies have shown that polyphenol-mediated changes in microbial composition and function contribute to the alleviation of colitis and colitis-associated cancer (CAC) (10). Furthermore, polyphenols have been shown to inhibit disease progression in non-alcoholic fatty liver disease, obesity, and cancer by restoring metabolic homeostasis and reshaping metabolic pathways (11). In the digestive tract, ingested curcumin ameliorated dysbiosis, impacted hepatic metabolic performance, and improved the amino acid metabolism-related pathways (12). Polyphenols from walnut septum have also been reported to exert anti-colitis effects by upregulating certain microbial genes involved in the metabolism of amino acids (13). Zhu et al. (14) further confirmed that oral polyphenol-nanozyme-armored probiotics boosted the improvement of IBD by inhibiting the activation of branched-chain amino acid synthesis. However, the effects of millet polyphenols on colitis and their regulatory role in amino acid metabolism remain unclear.

Foxtail millet (FM) bran, a by-product from FM to polished millet, has been widely reported for its antioxidant, anti-tumor, and immunomodulatory activities (15, 16). In a previous study, we extracted from FM bran and demonstrated that bound polyphenol of the inner shell (BPIS) could ameliorate colitis and CAC by remodeling the gut microbiome and alleviating mucosal barrier dysfunction (16–18). Furthermore, it was found that BPLP, which consists of 6 MW < 200 Da compounds (4-hydroxybenzoic acid, MW: 138; vanillic acid, MW: 168; syringic acid, MW: 198; p-coumaric acid, MW: 166; ferulic acid, MW: 194; and isoferulic acid, MW: 194), was a major active component of BPIS. Nevertheless, the precise mechanism underlying BPLP’s effects on colitis and metabolic disorders remains incompletely understood. In this study, a chronic colitis mouse model was constructed by DSS induction. The effects of BPLP on chronic colitis were evaluated through clinical assessments, inflammatory cytokine analysis, and evaluation of intestinal barrier tight junction proteins. The impact of BPLP on the metabolic profile of colitis mice was analyzed through fecal non-targeted metabolomics. Our results demonstrated that BPLP attenuated chronic colitis by regulating amino acid metabolism. These findings substantiate the potential of BPLP as a natural, low-toxicity candidate for IBD prevention and treatment, offering a novel perspective for investigating polyphenol mechanisms in colitis treatment through metabolic regulation. Furthermore, anti-inflammatory active polyphenols found in millet bran supported the dietary recommendations proposed by nutritionists that increasing whole grain intake brings great public health benefits.

## 2 Materials and methods

### 2.1 Chemicals and reagents

FM bran used for BPLP extraction was provided by Shanxi Qin-Zhou-Huang Millet (Group) Co., Ltd. (Changzhi, China). DSS was obtained from MP Biomedical (America). Kits of the enzyme-linked immunosorbent assay (ELISA) were obtained from Andy Gene Biotechnology Co., Ltd. (Beijing, China). Staining reagents and antibodies were provided by Servicebio Technology Co., Ltd. (Wuhan, China).

### 2.2 Preparation of BPLP

First, the BPIS was extracted according to the method of a previous study (11). Then, the BPIS was dissolved with methyl alcohol, detected using ultra-performance liquid chromatography-triple time-of-flight (TOF) mass spectrometer (UPLC-Triple-TOF/MS) (Waters, MA), and visualized using Waters Empower software (Waters, MA). The phenolic components of BPIS have been previously reported and are listed in Appendix-Table 1 (16). To obtain BPLP, the BPIS solution after dialysis desalination was sealed in a 200 Da dialysis bag and placed in distilled water to allow the phenols with a molecular weight less than 200 to separate out. Then, the distilled water was rotary evaporated and freeze-dried to obtain a powder form of BPLP, which consists of six compounds: 4-hydroxybenzoic acid, MW: 138; vanillic acid, MW: 168; syringic acid, MW: 198; p-coumaric acid, MW: 166; ferulic acid, MW: 194; and isoferulic acid, MW: 194.

### 2.3 Animal study

A total of 30 healthy male C57BL/6 J mice (6 to 8 weeks old) were obtained from Vital River Laboratory Animal Technology Co., Ltd., and raised in a specific pathogen-free (SPF) room with a temperature of 26 ± 1 °C, a humidity of 60 ± 5%, and a 12-h light/dark cycle in the laboratory animal service center of the China Institute for Radiation Protection. All experimental procedures were approved by the Committee on the Ethics of Animal Experiments of Taiyuan Normal University (Shanxi, China).

**Figure 1:**
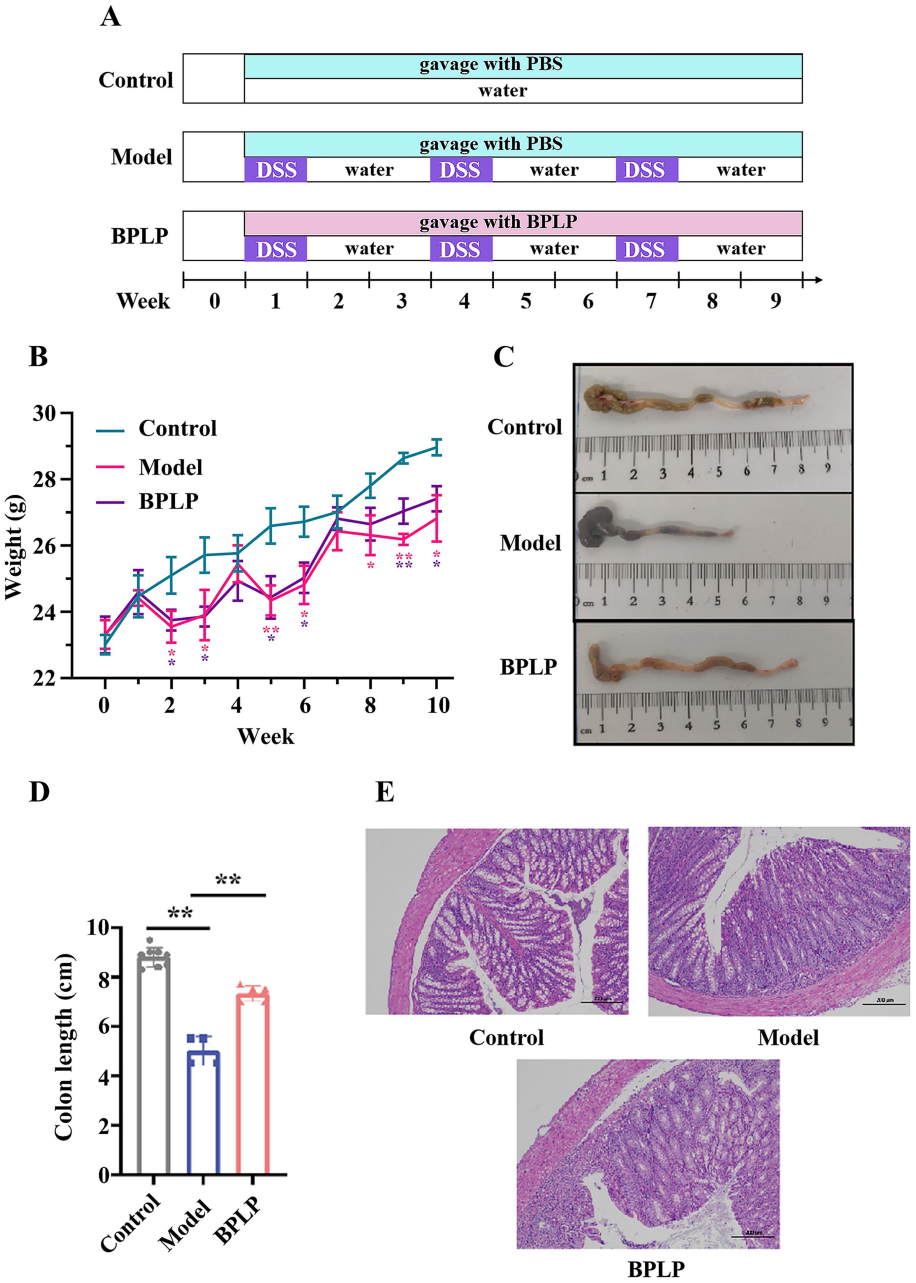
Effect of BPLP treatment on DSS-induced mice. **(A)** Schematic overview of the experimental design. **(B)** Average weight change of three groups. **(C)** The representative colon length from mice in the control, model, and BPLP groups. **(D)** Average colon length of different groups. **(E)** The pathological section of the colon tissues from mice in the control, model, and BPLP groups. Data represented as means ± SD (*n* ≥ 5); *p < 0.05; **p < 0.01.

As shown in Figure 1A, 1 week after acclimation, all mice were weighed and randomly divided into three groups: control (C), model (M), and BPLP (*n* = 10 in each group). The mice in the control group were not subjected to any treatment; the mice in the model group were administered intermittent 3 weeks of 2% DSS; and the mice in the BPLP group were administered DSS and gavaged with BPLP based on mouse weight (75 mg/kg). The mouse weight was detected once a week. After dissection, colonic length was measured, and colons were immobilized in 4% neutral formalin for 24 h for staining analyses.

### 2.4 Histological examination and immunohistochemistry staining

For histological analysis, colons, hearts, livers, spleens, lungs, kidneys, and pancreases were fixed, embedded, and sectioned. Then, they were stained with hematoxylin–eosin (H&E). For the immunohistochemistry (IHC) assay, the paraffin section was dewaxed to water and subjected to antigen retrieval, endogenous peroxidase blocking, and serum blocking. Then, claudin-1, occludin, and ZO-1 antibodies and corresponding species-specific secondary antibodies were separately added for incubation. After DAB staining and nuclear counterstaining, the sections were dehydrated and mounted. Finally, the staining results were examined and photographed with a microscope.

### 2.5 Enzyme-linked immunosorbent assay (ELISA)

For ELISA analysis, the blood sample was centrifuged, and the sediment was removed. The serum was than collected and stored in the −80 °C refrigerator. To test the concentrations of cytokines, ELISA kits (tumor necrosis factor-α (TNF-α), interleukin-1β (IL-1β), interleukin-6 (IL-6), and interleukin-10) were obtained from Andy Gene Biotechnology Co., Ltd. (Beijing, China), and the detection process was proceeded according to the manufacturer’s protocol.

### 2.6 Untargeted metabolomic analysis

Metabolic profiling of mouse feces was conducted by BIOTREE Biological Technology Co., Ltd. (Shanghai, China). Briefly, samples and extracting solutions containing acetonitrile, methanol (acetonitrile:methanol = 1:1, V/V), and an isotopically labeled internal standard mixture were mixed evenly in a ratio of 1:4, and then the mixtures were vortexed (40 s) and sonicated (15 min) in an ice water bath. Finally, stationary incubation was conducted at −40 °C to precipitate proteins. After 2 h, the mixtures were centrifuged at 13,000 *g* for 15 min at 4 °C, and the supernatants were collected and analyzed using LC–MS/MS.

LC–MS/MS analysis was performed by UHPLC with a BEH amide column (2.1 mm × 100 mm, 1.7 μm) system (Thermo Fisher) coupled to a QExactive HFX mass spectrometer (Thermo). Ammonium acetate and ammonium hydroxide solution (A) and acetonitrile (B) were prepared as the mobile phase. QE HFX mass spectrum acquisition software (Xcalibur, Thermo) was used to control sample injection automatically, the temperature was set at 4 °C, and the sample volume was 2 μL. Then, a full-scan mass spectrum was collected and continuously evaluated. Electrosprayionization (ESI) source circumstances are similar to those of Dunn et al. studies with some modifications (19). The conditions were as follows: “sheath gas flow rate of 30 Arb, aux gas flow rate of 25 Arb, capillary temperature of 350 °C, full MS resolution of 60,000, MS/MS resolution of 7,500, collision energy of 10/30/60 in NCE mode, and spray voltage of 3.6 kV (positive) or −3.2 kV (negative).” The modified condition was added to the revised manuscript.

### 2.7 Multi-omic bioinformatic analysis of IBD patients from PRISM and HMP2 datasets

The differential abundance of amino acids and their related metabolites in diseases of IBD conditions was evaluated with two public IBD metabolomic datasets from the Prospective Registry in IBD Study at MGH (PRISM) (20) and the IBDMDB (21) study within the Integrative Human Microbiome Project (HMP2) (22). The PRISM is a referral center-based, prospective cohort of IBD patients with CD (*n* = 68) and UC (*n* = 53) and healthy volunteers (*n* = 34). The metabonomics of the PRISM cohort were derived from supporting information in relevant publications. The IBDMDB HMP2 provided an integrated resource of the gut microbial ecosystem from 67 participants with CD, 38 participants with UC, and 27 healthy people. Profiles for HMP2 metagenomes were downloaded from http://ibdmdb.org.

### 2.8 Statistical analysis

For principal component analysis (PCA), using SIMCA software (V16.0.2, Sartorius Stedim Data Analytics AB, Umea, Sweden), the data underwent logarithmic transformation and CTR formatting, followed by automated modeling. PCA was visualized at http://www.bioinformatics.com.cn/. To conduct orthogonal partial least squares discriminant analysis (OPLS-DA), the data were processed using SIMCA with logarithmic transformation and UV formatting. First, OPLS-DA was performed on the first principal component, and the model quality was tested using 7-fold cross-validation. Then, the model effectiveness was evaluated using the *R*^2^*Y* (model interpretability for categorical variable *Y*) and *Q*^2^ (model predictability) obtained after cross-validation. Finally, a permutation test was used to further test the model’s effectiveness by randomly changing the order of the categorical variable *Y* multiple times to obtain different random *Q*^2^ values. All results were expressed in the mean ± standard deviation (SD) form. Single-variable comparisons were completed with a *t*-test using SPSS software. *p* < 0.05 indicated a significant difference, and *p* < 0.01 indicated an extremely significant difference.

## 3 Results

### 3.1 Effects of BPLP treatment on DSS-induced mice

**Figure 2:**
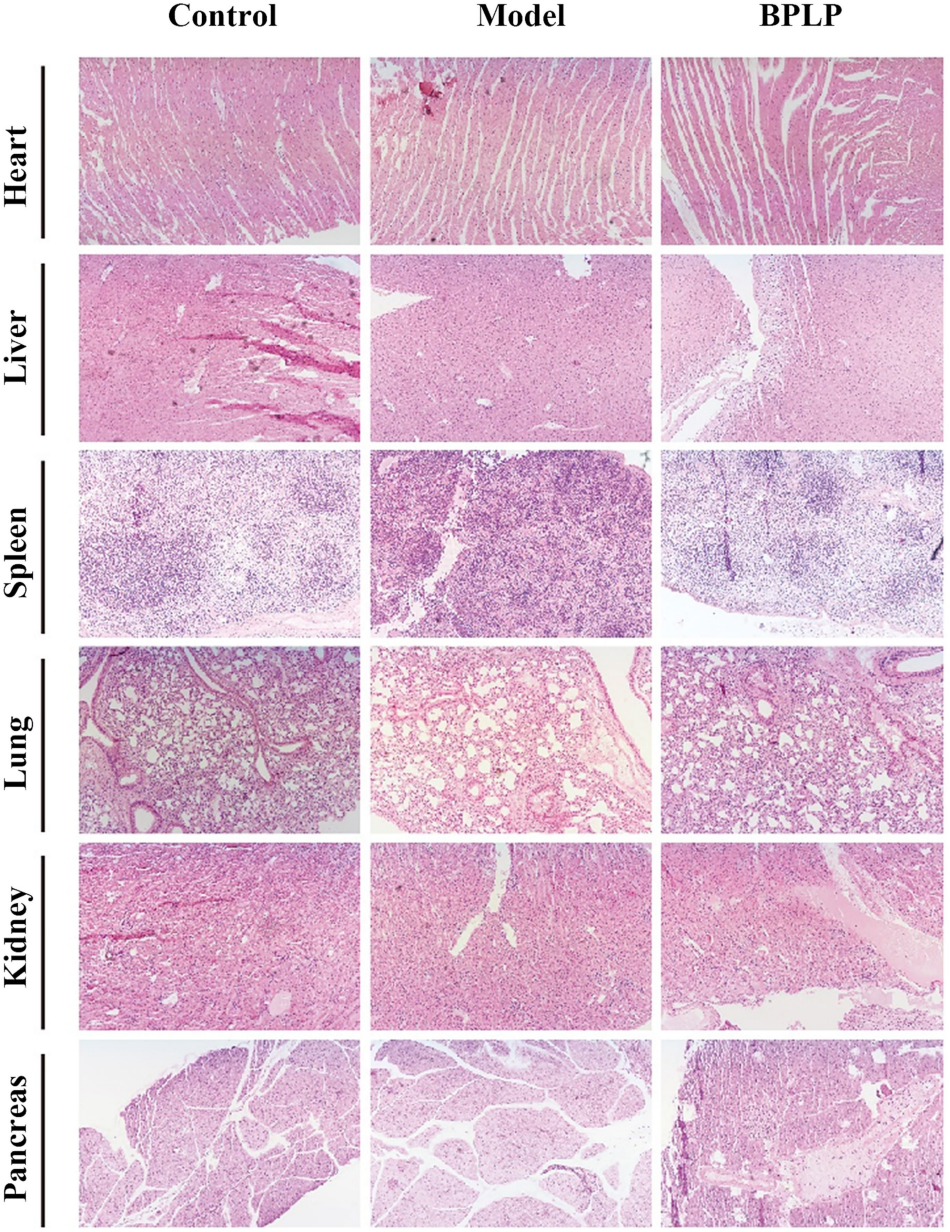
Representative H&E-stained slices of major organs (heart, liver, spleen, lung, kidney, and pancreas) from mice in three groups.

DSS-induced mice are widely used as a model of colitis. To estimate the anti-inflammatory activity of BPLP *in vivo*, the mice were administered three cycles of 2% DSS to construct chronic inflammation. BPLP was administered by gavage to mice at 75 mg/kg every day (Figure 1A). The results showed that the weight of the model group was diminished obviously compared to the control mice, while treatment with BPLP effectively prevented the weight loss (Figure 1B). As shown in Figures 1C,D, the length of the model group mouse colon was suggestively shorter than that of the control and BPLP groups (control group: 8.81 ± 039 cm, model group: 5 ± 0.57 cm, and BPLP group: 7.34 ± 0.32 cm). Histopathological results showed that crypt distortion and inflammatory infiltration significantly appeared in the mice from the model group. Furthermore, the colons of the control and BPLP groups showed more surface epithelium and complete crypts than those of the model group (Figure 1E). H&E-stained sections also confirmed that supplementation with BPLP had no toxic damage to the heart, liver, spleen, and other major organs (Figure 2). In short, these results indicated that BPLP effectively alleviated the inflammatory response in DSS-induced chronic colitis mice.

### 3.2 BPLP decreases the level of inflammatory cytokines

**Figure 3:**
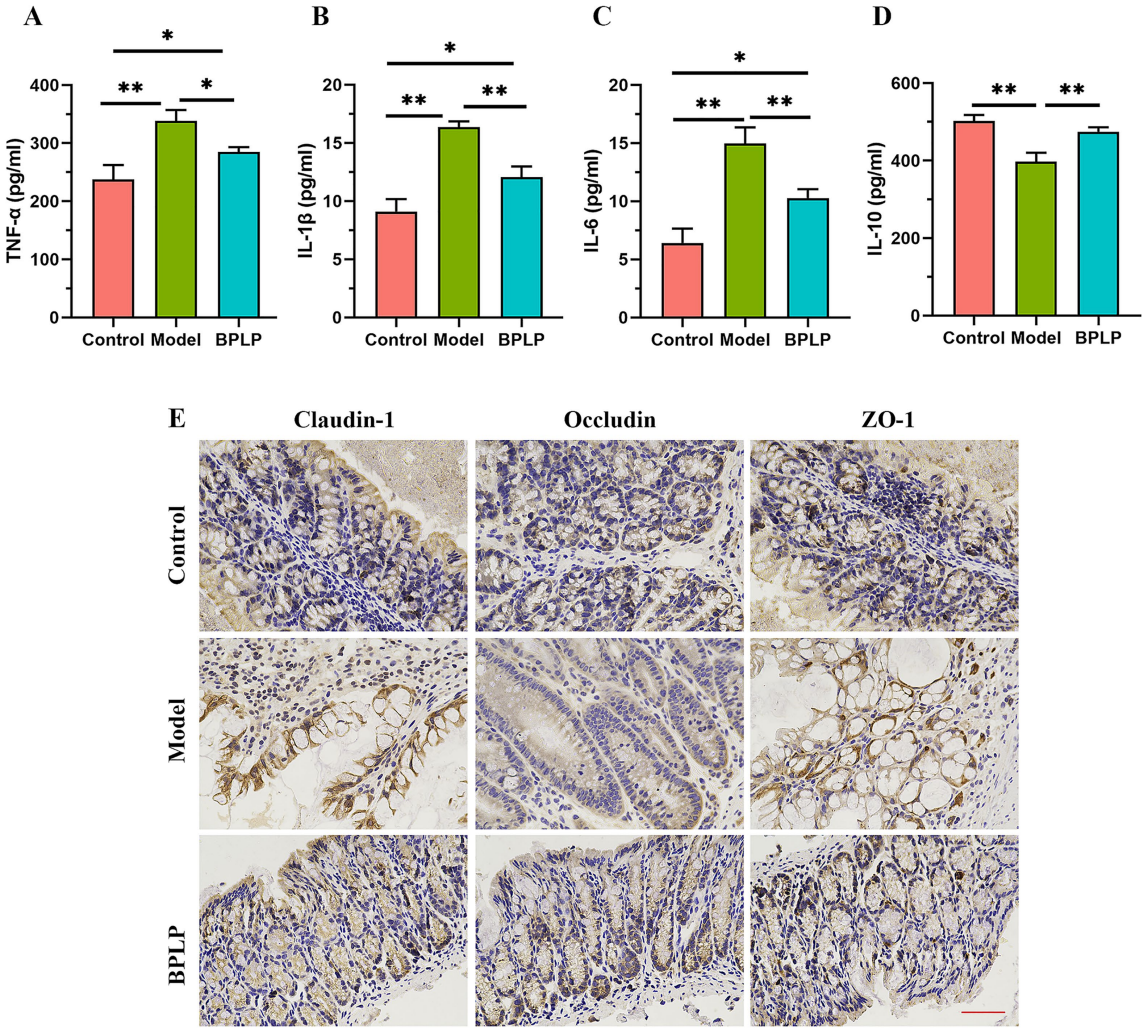
BPLP suppressed inflammatory cytokines and inhibited the loss of epithelial barrier integrity. **(A–D)** Concentrations of the cytokines TNF-α **(A)**, IL-1β **(B)**, IL-6 **(C)**, and IL-10 **(E)** were determined by ELISA kit. **(E)** Representative IHC staining images of tight junction proteins claudin-1, ZO-1, and occludin. Scale bars, 100 μm. Data represented as means ± SD (*n* ≥ 5); *p < 0.05; **p < 0.01.

To assess the influence of BPLP on chronic colitis, the concentrations of pro-inflammatory cytokines TNF-α, IL-1β, and IL-6 were detected using the ELISA assay. The results showed that drinking DSS water caused increases in TNF-α, IL-1β, and IL-6 compared to the control mice; however, supplementation with BPLP reduced the augmentation of the above inflammatory factors (Figures 3A–C). Moreover, compared with the model mice, the concentration of the anti-inflammation factor IL-10 was markedly increased in the control and BPLP groups (Figure 3D). The destruction of colonic barrier integrity giving rise to the invasion of pathogens and toxins is an important factor for triggering and aggravating inflammation. In colons, the physical barrier consisting of tight junction proteins is vital in the anti-inflammation reaction and immunoregulation. To assess the consequence of BPLP on the gut barrier, the expressions of tight junction proteins, including claudin-1, occludin, and ZO-1, were tested by IHC staining. It was found that the levels of tight junction proteins declined in the mice of the model group, while remarkable retrieval was observed in BPLP-treated colitis mice (Figure 3E). Overall, these results showed that BPLP maintained intestinal epithelial barrier function and inhibited the secretion of inflammatory factors.

### 3.3 BPLP treatment reformed metabolite profiling in chronic colitis

**Figure 4:**
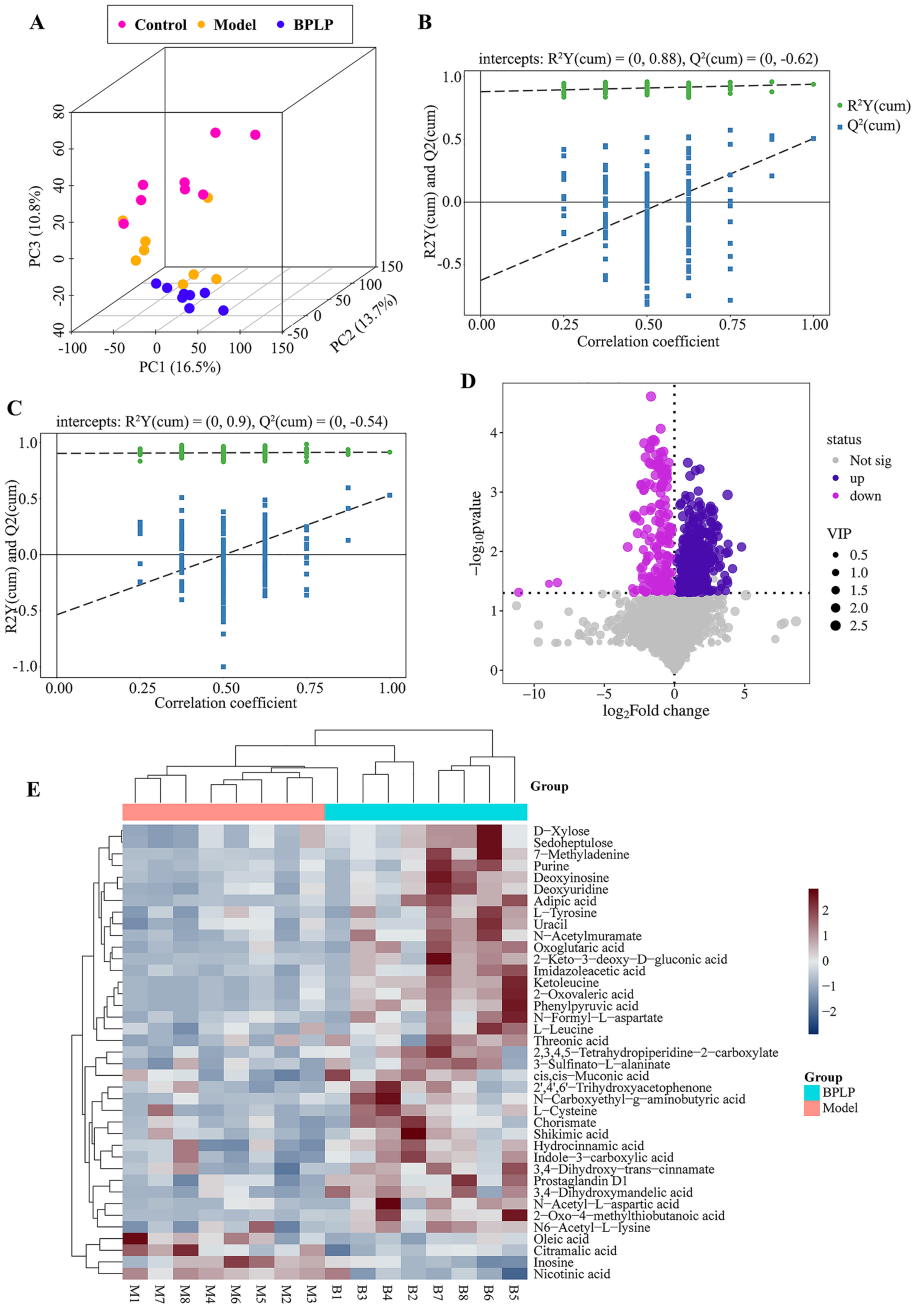
Altered metabolites related to the treatment of BPLP. **(A)** Principal component analysis (PCA) of samples from the three groups. **(B,C)** Permutation test of the OPLS-DA model for groups control vs. model **(B)** and BPLP vs. model **(C)**. **(D)** Volcanic map of differential metabolites between the BPLP and model groups. **(E)** Cluster heat maps of the top forty differential metabolites between the BPLP group and the model group.

To further systematically investigate the mechanism by which BPLP alleviates colitis, LC–MS analysis was executed on the feces of mice. The PCA disclosed that the samples from three groups were significantly separated, respectively (Figure 4A), indicating that the metabolism of mice was disordered remarkably after drinking DSS, while administration of BPLP affected the alteration of the metabolic profile. Then, we adopted the OPLS-DA method for further screening of potential markers. VIP values of ≥1.0 and *p*-values of <0.05 were used to filter the difference between the two groups to the greatest extent. Through the permutation test, we casually reformed the arrangement of *Y* and established the corresponding OPLS-DA model several times to obtain random R2 and Q2 values. As shown in Figures 4B,C, the R2Y of the OPLS-DA simulation between each group was greater than 0.4, and the intercept of the linear equation after refitting the Q2 value in the replacement test was all less than −0.1, indicating that the OPLS-DA model had great predictability and no overfitting phenomenon. This evidence indicated that BPLP could regulate the metabolic disorder of colitis mice.

### 3.4 Treatment with BPLP altered amino acid metabolites and related pathways

To explore the different metabolites that cause the metabolic profile changes in the BPLP intervention mice, VIP values of ≥ 1 and *p*-values of ≤0.05 from validated OPLS-DA models were seen as the screening conditions of different metabolites. The results showed that 695 different metabolites were identified. Figure 4D shows the significantly changed metabolites between the BPLP and model groups. Furthermore, using |LogFC| ≥ 2 as the selection condition, it was found that 97 metabolites were significantly elevated and 32 metabolites were markedly decreased after BPLP treatment. Next, we analyzed the top forty metabolites with significant changes between the BPLP and model groups. The data showed that the contents of oleic acid, citramalic acid, inosine, and nicotinic acid were significantly decreased, while metabolites such as purine, L-tyrosine, and L-leucine were significantly increased in the BPLP group.

**Figure 5:**
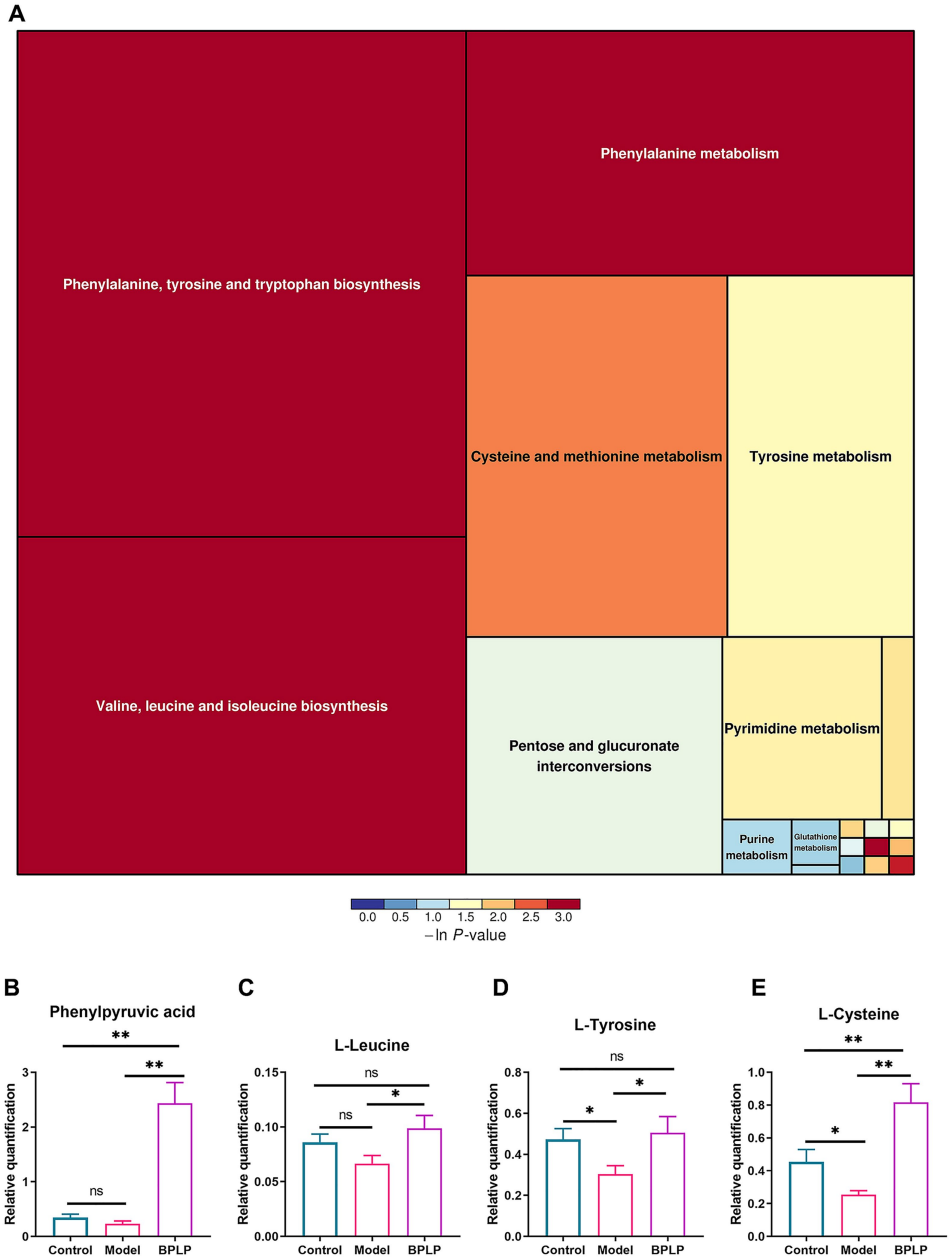
Metabolite pathway adjusted by BPLP in chronic colitis. **(A)** KEGG enrichment analysis of differential metabolites between the BPLP group and the model group. **(B–E)** Relative quantitative statistics of differential metabolites, including phenylpyruvic acid **(B)**, leucine **(C)**, tyrosine **(D)**, and cysteine **(E)** under enriched signaling pathways. Data represented as means ± SD (*n* ≥ 5); *p < 0.05; **p < 0.01.

To further thoroughly examine the pathway by which BPLP inhibits the inflammatory effect in colitis mice, we annotated the altered metabolites with the KEGG database^1^ to regulate the position and roles of each compound in a particular metabolic pathway. The data presented that a series of metabolic pathways related to differential metabolites were enriched. The intersection of the changed metabolic pathways in all groups further found that amino acid-related signaling pathways, including phenylalanine/tyrosine/tryptophan biosynthesis, valine/leucine/isoleucine biosynthesis, phenylalanine metabolism, and cysteine and methionine metabolism, were significantly affected by BPLP (Figure 5A). As expected, the concentrations of amino acids such as phenylpyruvic acid, L-tyrosine, L-leucine, and L-cysteine were remarkably elevated (Figures 5B–E). These amino acids have all been linked to inflammation in the gut.

### 3.5 Changed metabolites in BPLP therapy colitis mice were consistent with clinical IBD patient datasets

**Figure 6:**
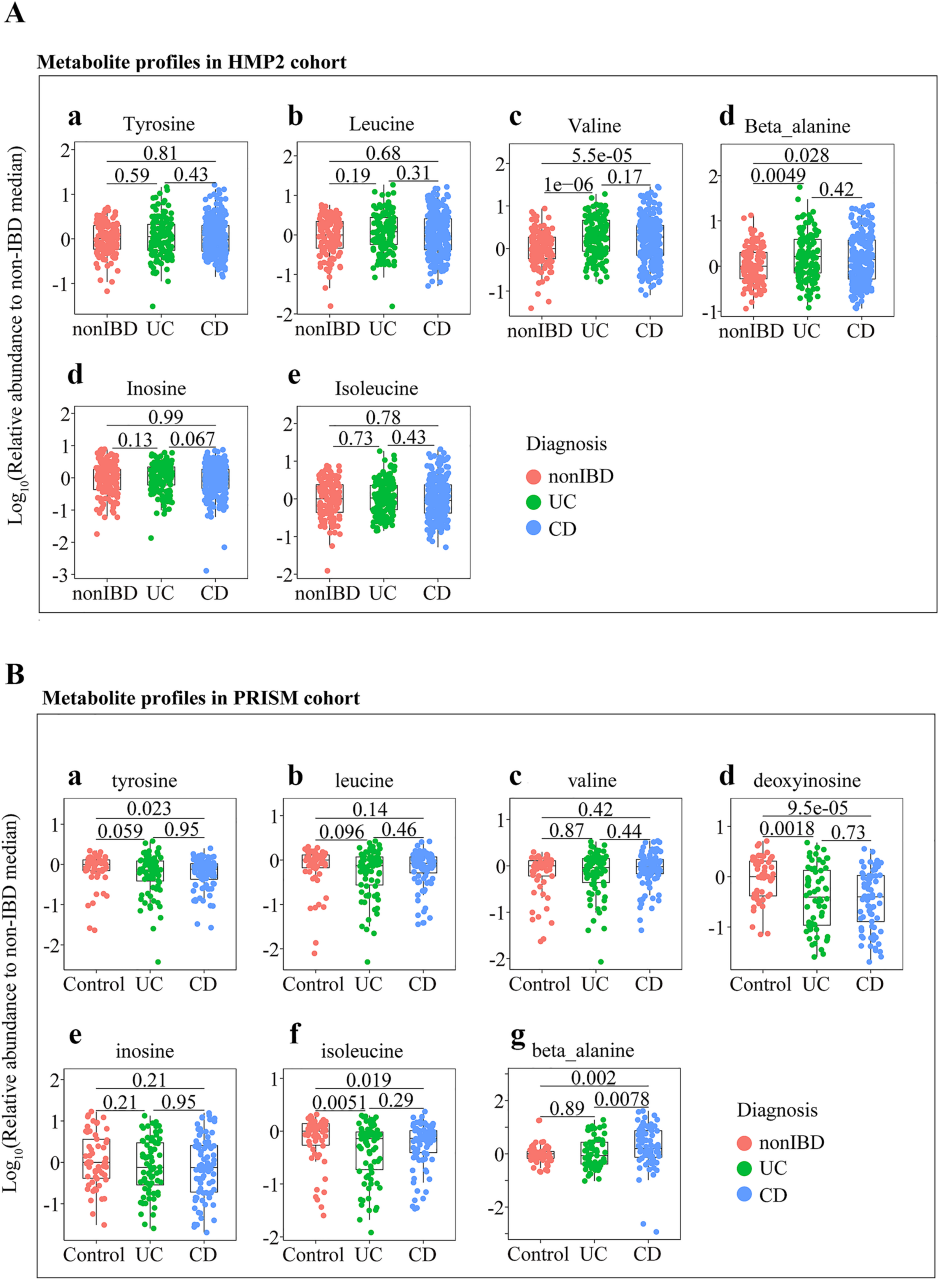
Changes in metabolites associated with BPLP therapy in healthy and colitis patients. **(A)** Levels of amino acid and metabolite detected in the HMP2 cohort. **(B)** Levels of amino acid and metabolite detected in the PRISM cohort.

Next, to further verify the trends of the above different metabolites affected by BPLP in clinical patients, two publicly available IBD metabolomic datasets were used, including the PRISM (20) and the IBDMDB HMP2 (21). As shown in Figure 6, compared to healthy people, the abundances of tyrosine, deoxyinosine, and isoleucine were notably reduced in IBD patients, while the abundances of valine and β-alanine were obviously increased in UC and CD patients (*p* < 0.05). The trend of these differential metabolites was consistent with the trend we have shown above in the control, model, and BPLP groups. These data indicated that amino acids and their metabolites were closely related to the development of colitis.

## 4 Discussion

IBD has drawn global attention due to its high incidence, difficulty in radical treatment, and easy recurrence (23). As one of the significant natural active substances, polyphenols have been extensively studied in preventing and treating colitis and related diseases. Previous studies have shown that BPLP is an active component of BPIS. The present findings further revealed that supplementation with BPLP significantly ameliorated chronic colitis by restoring weight, intestinal length, and other colitis-related phenomena of mice. Our results further supported that polyphenols protect against IBD in numerous distinct manners. The gut epithelial barrier plays a crucial role in preventing harmful antigens and microorganisms and assuring the absorption of nutrients and energy (24). In a disease state, the equilibrium between the environment and the internal milieu of the body is disrupted, leading to increased intestinal barrier permeability and the penetration of inflammatory cytokines. The tight junction complex consists of multiple proteins that serve as the regulation of paracellular permeability and control the passage of antigens. In our study, although the specific mechanisms still need to be investigated, there are sufficient data to show that after administration, BPLP significantly upregulated the expression of tight junction claudin-1, occludin, and ZO-1 proteins; maintained the integrity and permeability of the intestinal barrier; and suppressed the increase of inflammatory factors TNF-α, IL-1β, and IL-6.

A large amount of clinical evidence shows that pathophysiological disorders in the process of IBD are associated with significant changes in metabolomics. Kolho et al. (25) found that compared with healthy people, amino acid metabolism, folic acid biosynthesis, and signaling pathways were significantly interfered with in the serums of patients with Crohn’s disease. Nikolaus et al. (6) found that tryptophan deficiency was closely related to the occurrence of IBD. Considering the metabolic disorder in IBD patients and the effect of polyphenols on metabolism, we performed non-target metabolomic analysis of mouse feces. OPLS-DA analysis reflected the variation of metabolites in fecal samples between colitis mice and healthy controls. A total of 147 differential metabolites were affected by BPLP supplementation, and they were mainly enriched in pathways involved in amino acid metabolism, including phenylalanine/tyrosine/tryptophan biosynthesis, valine/leucine/isoleucine biosynthesis, and phenylalanine metabolism. Next, we used two IBD metabolomic datasets, PRISM and IBDMDB HMP2, to determine the variation in the abundance of differential metabolites under disease ecological conditions and found the same trend.

Current studies have proven that amino acids play significant roles in intestinal inflammation (8). The relieving effect of amino acids on colitis may be thoroughly correlated to the expression of tight junction proteins, apoptosis and proliferation of intestinal epithelial cells, the NF-𝜅B signal pathway, and the nuclear erythroid-related factor 2 signaling pathway (26, 27). Valine, leucine, and isoleucine can enhance the intestinal immune defense system by improving intestinal morphological integrity and immunoglobulin production (28). Cysteine has been reported to act as an active substance targeting redox switches in proteins, thereby affecting IBD by influencing gene regulation, DNA damage, ion transport, intermediate metabolism, and mitochondrial function (29). Studies have found that in pediatric CD patients’ feces, Cys significantly increased after receiving anti-tumor necrosis factor treatment or dietary therapy (30). Tryptophan has been publicized to augment tight junction protein’s expression in the intestine of pigs (31, 32). 6-Hydroxymelatonin, a metabolite of tryptophan, has been shown to have antioxidant activity. Furthermore, the concentrations of serum tryptophan in IBD patients were lower than those in healthy controls (6, 33). Phenylalanine can inhibit the generation of TNF-α and enhance immune response in the treatment of IBD (34). Tyrosine is an essential component of protein synthesis and immune response and also has a beneficial effect on intestinal inflammation (34). Tryptophan, phenylalanine, and tyrosine have been shown to decrease colonic inflammation in piglets by activating CaSR (35). These findings suggested that alteration of amino acids regulated by BPLP plays an indispensable part in relieving colitis.

Evidence has revealed the intricate connection between microbiome and its metabolites and the development of IBD (36). More than 200 risk variants exist in the human genome that drive the development of IBD, many of which are responsible for host–microbe interactions (21). In the intestine, tryptophan metabolism pathways producing serotonin, kynurenine, and indole derivatives are confirmed to be directly or indirectly affected by microbiota (37). Some commensal microbes, including *Lactobacillus* and *Bifidobacterium,* can convert tryptophan to the intermediate indole-3-lactic acid (38), which is further converted to indole propionic acid by bacteria such as *Clostridium* and *Peptostreptococcus* (39, 40). In addition to the influence of amino acid metabolism, the gut microbiome plays a vital role in the synthesis of amino acids. Several *in vitro* studies have shown that bacteria such as *Prevotella bryantii, Selenomonas ruminantium,* and *Streptococcus bovis* participate in *de novo* synthesis of amino acids under certain conditions (41, 42). Our previous studies have indicated that BPLP could ameliorate colitis and CAC by remodeling the gut microbiome, including *Lachnospiraceae, Rikenellaceae, and Prevotella.* These findings suggested that the microbiota might mediate the regulation of amino acid metabolism and colitis alleviation in colitis mice supplemented with BPLP, whose mechanism needs more studies to explore. Notably, IBD is a disease of chronic immune-mediated disorders in which patients amplify and sustain local or systemic inflammation. Tryptophan derivatives indole and kynurenine serve as agonists to stimulate the aryl hydrocarbon receptor to promote the T regulatory cell differentiation from naive T cells and confine Th17 and Th1 responses, thereby inhibiting inflammation (43). Natural seleno-amino acids have been proven to reverse decreased glutathione peroxidase 4, protect against intestinal epithelial cell injury, and reduce immune cell infiltration in IBD (44). Therefore, when further focusing on exploring the regulatory effects of BPLP in amino acid metabolism in the gut microbiota, the influence of polyphenol and its regulated specific microbes on the host’s immune response is a non-negligible factor.

In summary, in DSS-induced chronic colitis, BPLP effectively and safely attenuates the inflammation, inhibits inflammatory factors, and protects against the integrity of the intestinal barrier. In the intestine, BPLP regulates amino acids and related metabolites, affecting metabolism signaling pathways including phenylalanine/tyrosine/tryptophan biosynthesis, valine/leucine/isoleucine biosynthesis, and phenylalanine metabolism. These findings provide valuable strategies for disclosing the mechanism of polyphenol in the prevention and treatment of colitis from the perspective of metabolomics.

## Data Availability

The original contributions presented in the study are included in the article/Supplementary material, further inquiries can be directed to the corresponding authors.

## Footnotes

1
https://www.kegg.jp/
