# Supplementary material for: Inhibitory effects and amino acid metabolism regulations of active polyphenol from foxtail millet bran on chronic colitis in mice
Source: Front Nutr. 2025 Dec 18;12:1714755. doi: 10.3389/fnut.2025.1714755 (PMC12756112; doi:10.3389/fnut.2025.1714755)
Supplement: Supplementary file 1 [file Table_1.docx]

**Appendix-Table 1 phenolic components of BPIS**

| No. | Phenolic Acids | molecular formula | molecular weight | Component |
| --- | --- | --- | --- | --- |
| 1 | 4-hydroxybenzoic acid | C_7_H_6_O_3_ | 138 | BPLP |
| 2 | p-coumaric acid | C_9_H_8_O_3_ | 166 |  |
| 3 | vanillic acid | C_8_H_8_O_4_ | 168 |  |
| 4 | ferulic acid | C_10_H_10_O_4_ | 194 |  |
| 5 | isoferulic acid | C_10_H_10_O_4_ | 194 |  |
| 6 | syringic acid | C_9_H_10_O_5_ | 198 |  |
| 7 | anillic acid 4-O-β-D-glucopyranoside | C_14_H_18_O_9_ | 330 | Synergy components |
| 8 | ferulic acid 4-O-β-  D-glucopyranoside | C_16_H_20_O_9_ | 356 |  |
| 9 | glucosyringic acid | C_15_H_20_O_10_ | 360 |  |
| 10 | 4,4′-dihydroxy-3,5′-dimethoxy,3′-bicinnamic acid | C_20_H_18_O_8_ | 386 |  |
| 11 | biferulic acid | C_20_H_18_O_9_ | 402 |  |
| 12 | vitexin | C_21_H_20_O_10_ | 432 |  |
